# Surgically resected primary esophageal choriocarcinoma accompanied with Barrett’s adenocarcinoma: a case report

**DOI:** 10.1186/s40792-020-00990-y

**Published:** 2020-09-29

**Authors:** Yuta Fujiwara, Koichi Okamoto, Itasu Ninomiya, Hiroto Saito, Takahisa Yamaguchi, Shiro Terai, Jun Kinoshita, Isamu Makino, Keishi Nakamura, Sachio Fushida, Hiroko Ikeda, Tetsuo Ohta

**Affiliations:** 1 Department of Gastroenterological Surgery, Kanazawa University, 13-1 Takara-Machi, Kanazawa, Ishikawa 920-8641 Japan; 2 Department of Diagnostic Pathology, Kanazawa University, 13-1 Takara-Machi, Kanazawa, Ishikawa 920-8641 Japan

**Keywords:** Choriocarcinoma, Barrett’s adenocarcinoma, Esophagectomy, Esophagus

## Abstract

**Background:**

Choriocarcinomas are usually classified as either gestational or non-gestational. Primary choriocarcinomas in the gastrointestinal tract, especially primary choriocarcinomas in the esophagus, are extremely rare. We report a case of a rare primary esophageal choriocarcinoma mixed with squamous cell carcinoma-like components in association with Barrett’s adenocarcinoma.

**Case presentation:**

A 58-year-old man visited the hospital, complaining of hematemesis and tarry stools. In emergency upper gastrointestinal endoscopy, a bleeding esophageal tumor was observed. Additionally, a contrast computed tomography (CT) scan showed a large hypervascular tumor 4.8 cm in diameter in the left kidney. He came to our institution for further examination and treatment of the esophageal tumor and kidney lesion. The patient had an easy bleeding elevated tumor 2 cm in diameter at the left wall of the middle thoracic esophagus and a left renal carcinoma. Histopathological diagnosis of the biopsy specimen of the esophageal tumor was a poorly differentiated carcinoma. However, a precise histological type diagnosis could not be obtained. In June 2016, mediastinoscopic transhiatal esophagectomy and posterior mediastinal gastric tube reconstruction were performed to treat his esophageal tumor. Histopathologically, most of the tumor comprised hCG-positive syncytiotrophoblasts. Therefore, we confirmed it as a primary esophageal choriocarcinoma. Furthermore, the tumor contained a poorly differentiated squamous cell carcinoma-like component that was also diagnosed as a choriocarcinoma using immunohistochemical staining and there was a small Barrett’s esophageal adenocarcinoma lesion in the Barrett's epithelium near the tumor. Three months after surgery, a CT scan demonstrated multiple lung metastatic nodules and multiple intrahepatic masses. Needle biopsy from the lung nodule showed a choriocarcinoma. Despite chemotherapy, the metastatic choriocarcinoma regrew rapidly and multiple bone metastases appeared. He died because of his esophageal choriocarcinoma 13 months after primary resection.

**Conclusions:**

We encountered an extremely rare case of esophageal choriocarcinoma combined with squamous cell carcinoma-like components in association with a simultaneous Barrett’s adenocarcinoma that we followed for the entire course of his disease, from resection to end of life. Esophageal choriocarcinomas are rare with peculiar characteristics and very poor prognoses. Additional cases are needed to establish an appropriate future treatment.

## Background

Choriocarcinomas often occur in the gonads, uterus, mediastinum and retroperitoneum [1]. Primary choriocarcinomas in the gastrointestinal tract are rare, especially primary esophageal choriocarcinomas, which are extremely rare [2–13]. We report the world’s first resected case of a primary esophageal choriocarcinoma combined with squamous cell carcinoma (SCC)-like components in association with Barrett’s adenocarcinoma.

## Case presentation

**Fig. 1:**
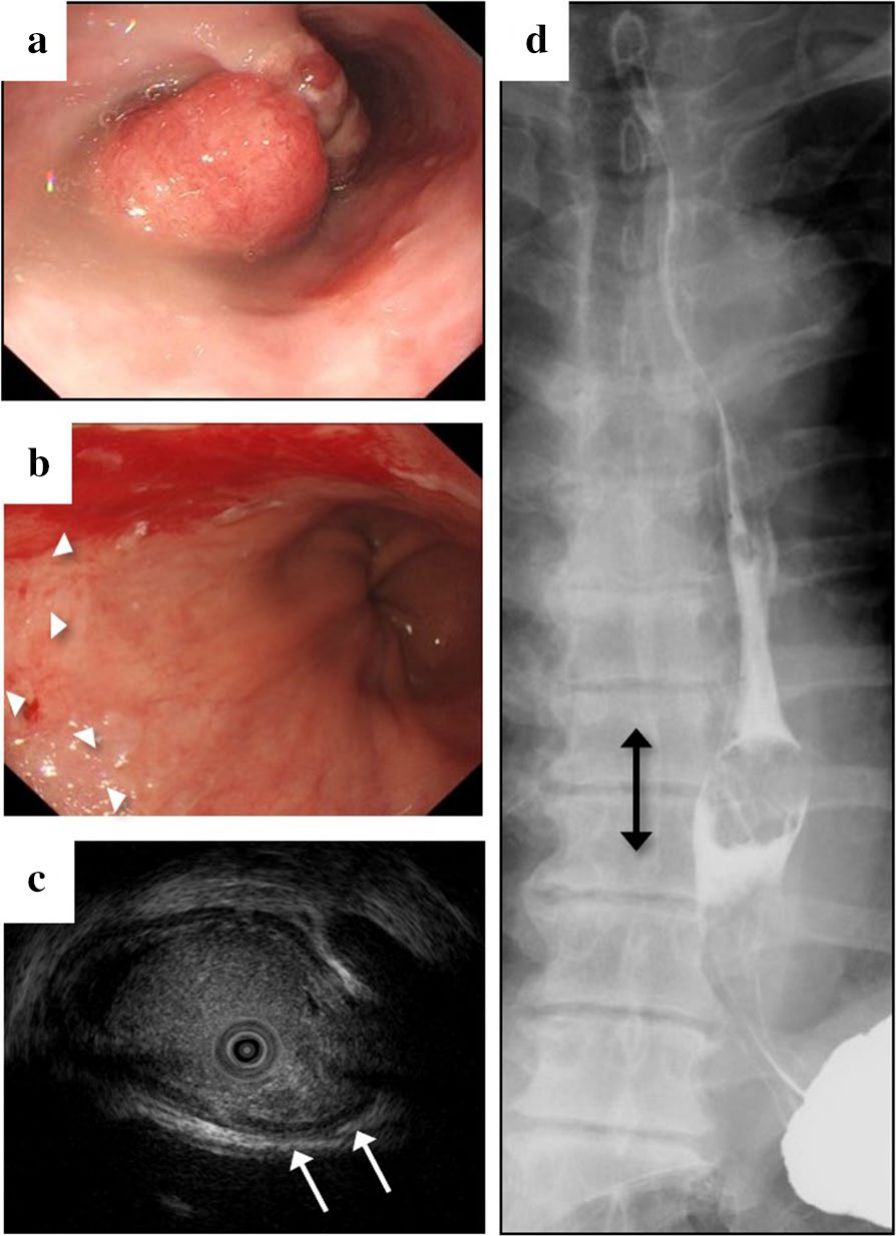
Upper gastrointestinal endoscopic and gastrointestinal contrast examination findings. A pendulous tumor approximately 2.0 cm in diameter was found at the left wall of the middle thoracic esophagus (**a**). Long segment Barrett’s esophagus extending about 4.0 cm in length was observed at the anal side of the tumor (arrowhead) (**b**). Endoscopic ultrasonography showed the tumor infiltrating into the deep submucosal layer (white arrow) (**c**). Gastrointestinal contrast examination showed a defect image of 2.2 cm in the lower thoracic esophagus (double-ended black arrow) (**d**)

**Fig. 2:**
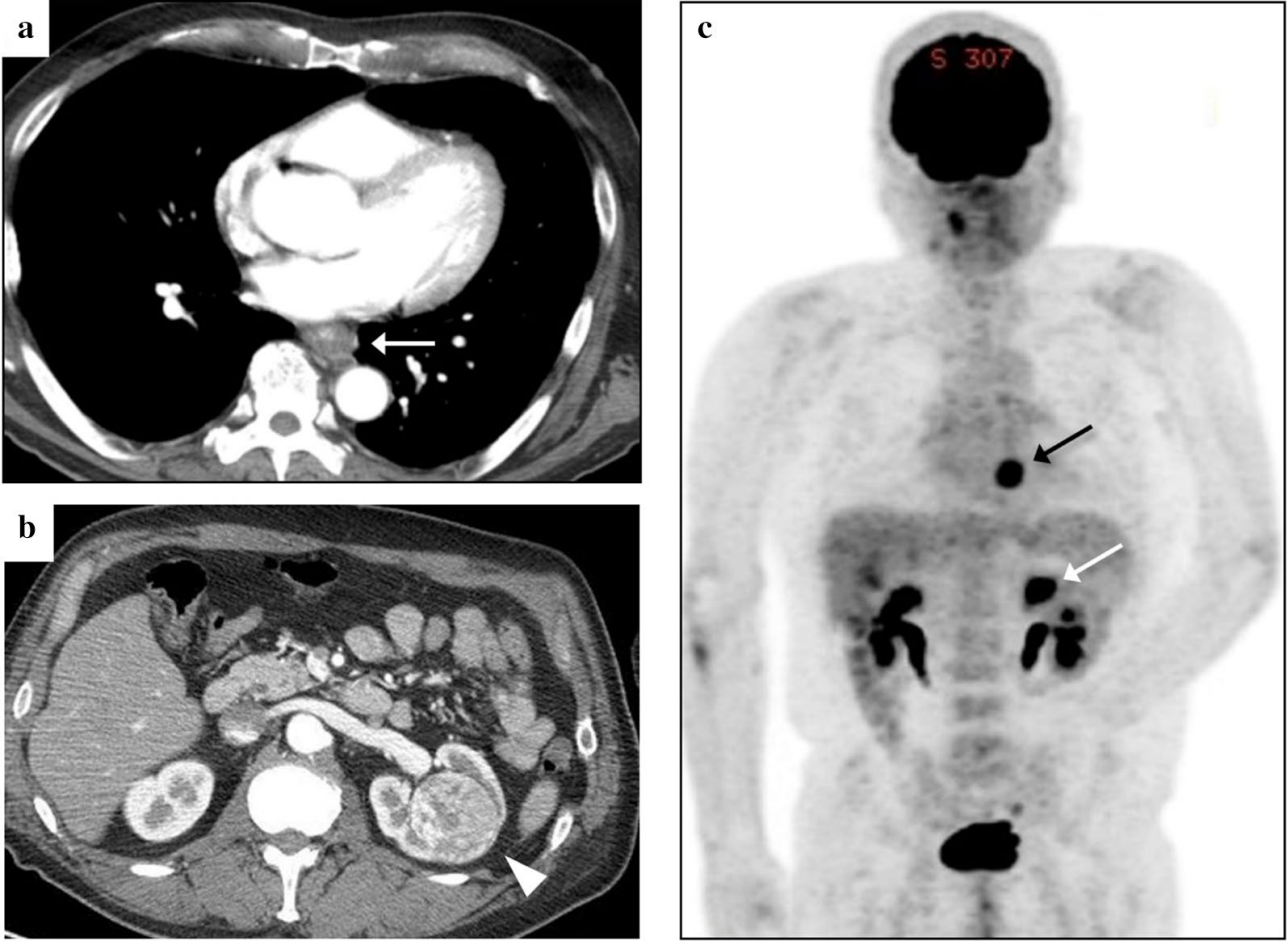
Enhanced CT examination showed a tumorous lesion occupying the esophageal lumen in the lower thoracic esophagus (arrow) (**a**). A 48-mm-diameter hypervascular tumor in the left kidney medulla (arrowhead) (**b**). High accumulation of 2-[^18^F] fluoro-2-deoxy-d-glucose (FDG) was observed in esophageal lesion and left kidney lesion (black and white arrow, respectively). There was no FDG accumulation indicating lymph node and distant organ metastasis (**c**)

A 58-year-old man, who had hypertension, hyperlipidemia and the past history of cerebral infarction and had past smoking and drinking history, visited his nearby hospital, complaining of hematemesis and tarry stools. As a family history, his mother had treated for colon cancer. Endoscopic examination showed a hemorrhagic esophageal tumor. Additionally, a contrast computed tomography (CT) scan showed a large hypervascular tumor of 4.8 cm in diameter in the left kidney. He came to our institution for further examination and treatment of the esophageal and kidney lesions. On blood examination, mild anemia of serum hemoglobin (12.7 mg/dl) was observed. SCC, carcinoembryonic antigen (CEA) and cytokeratin 19 fragment (CYFRA) were within normal range. Serum human chorionic gonadotropin (hCG) was not measured. Upper gastrointestinal endoscopy showed an easy bleeding elevated tumor of 2 cm in diameter at the left wall of the middle thoracic esophagus (Fig. 1a). Histopathological diagnosis from biopsied specimens was a poorly differentiated carcinoma. However, a precise diagnosis could not be obtained from the biopsy specimen. Long segment Barrett’s esophagus (LSBE) of about 4 cm in length was observed at the anal side of the tumor (Fig. 1b). Endoscopic ultrasonography showed a tumor with a combination of isoechoic and low echoic components infiltrating into the submucosal layer (Fig. 1c). Upper gastrointestinal contrast examination showed an elevated lesion of 2.2 cm in diameter in the middle thoracic esophagus (Fig. 1d). Contrast-enhanced CT examination showed the tumorous lesion occupying the esophageal lumen of the middle thoracic esophagus (Fig. 2a). Swollen mediastinal or abdominal lymph nodes and distant metastases were not observed. Additionally, a solid tumor of 4.8 cm in diameter was found in the left kidney medulla with hyperenhancement in the early phase and washout of contrast in the delayed phase (Fig. 2b). 2-[^18^F] fluoro-2-deoxy-d-glucose (FDG)-positron emission tomography (PET) examination showed abnormal FDG accumulation in the esophageal tumor. The maximum standardized uptake values (SUVmax) in early and delay phases were 12.7 and 15.7, respectively. There was no suspected metastatic accumulation in the mediastinal or abdominal lymph nodes and other organs. Another abnormal FDG accumulation was observed in the left kidney lesion which was diagnosed as a primary renal cancer by its typical image findings (Fig. 2c). He was diagnosed with esophageal cancer [MtLt 2.2 cm Type 0-Ip cT1b N0 M0 cStage I] combined with a left renal cancer. His general condition was poor (ECOG-Physical Status 2) because of a left hemi-paralysis caused by cerebral infarction and was given anticoagulating agent. We preferentially performed esophagectomy to control bleeding from esophageal tumor prior to renal cancer treatment. Therefore, mediastinoscopic transhiatal esophagectomy and posterior mediastinal gastric tube reconstruction was performed as a first surgery.

## Histopathological findings

**Fig. 3:**
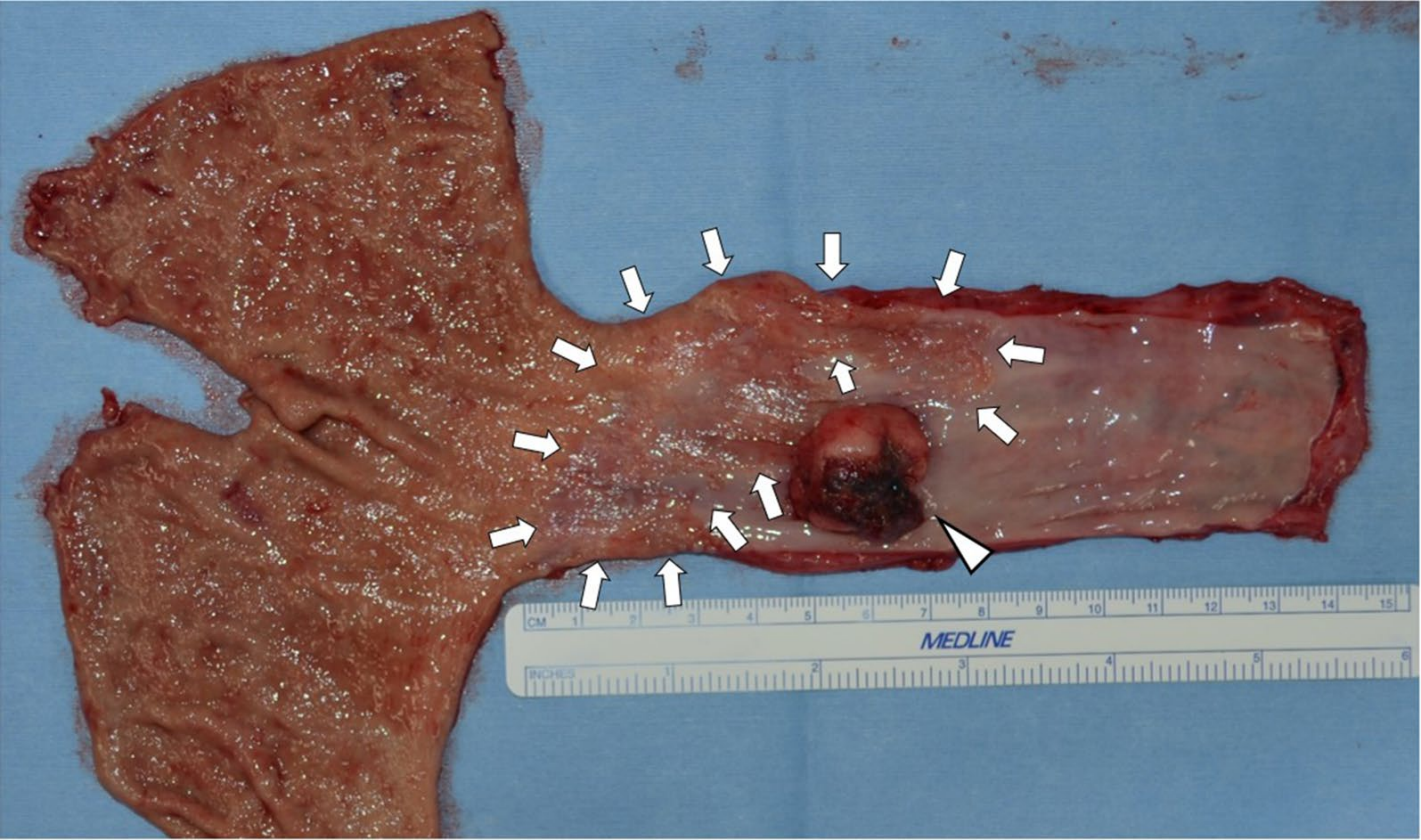
Macroscopic findings of the resected specimen. A 2.5- × 2.3- × 1.0-cm elevated tumor with clotting blood and necrotic tissues was found in the middle thoracic esophagus (arrowhead). Intestinal epithelium indicating Barrett’s esophagus was observed from the middle of the resected esophagus to the esophagogastric junction (arrow)

**Fig. 4:**
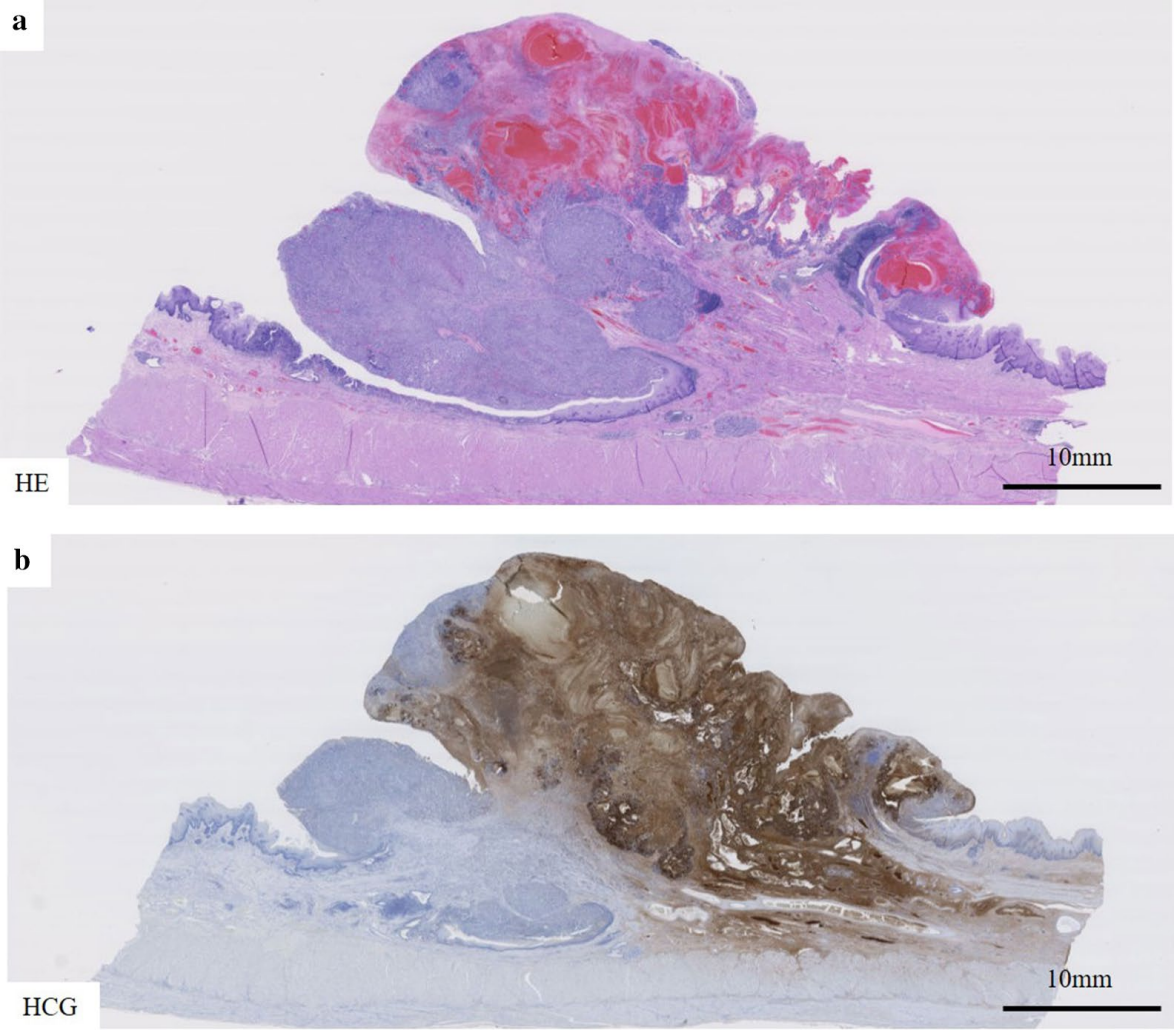
Microscopic findings of the choriocarcinoma (low magnification). The elevated tumor comprised a hemorrhagic portion and a medullary portion with small tumor cells (**a**). Most of the elevated tumor was positive for hCG using immunohistochemical staining (**b**)

**Fig. 5:**
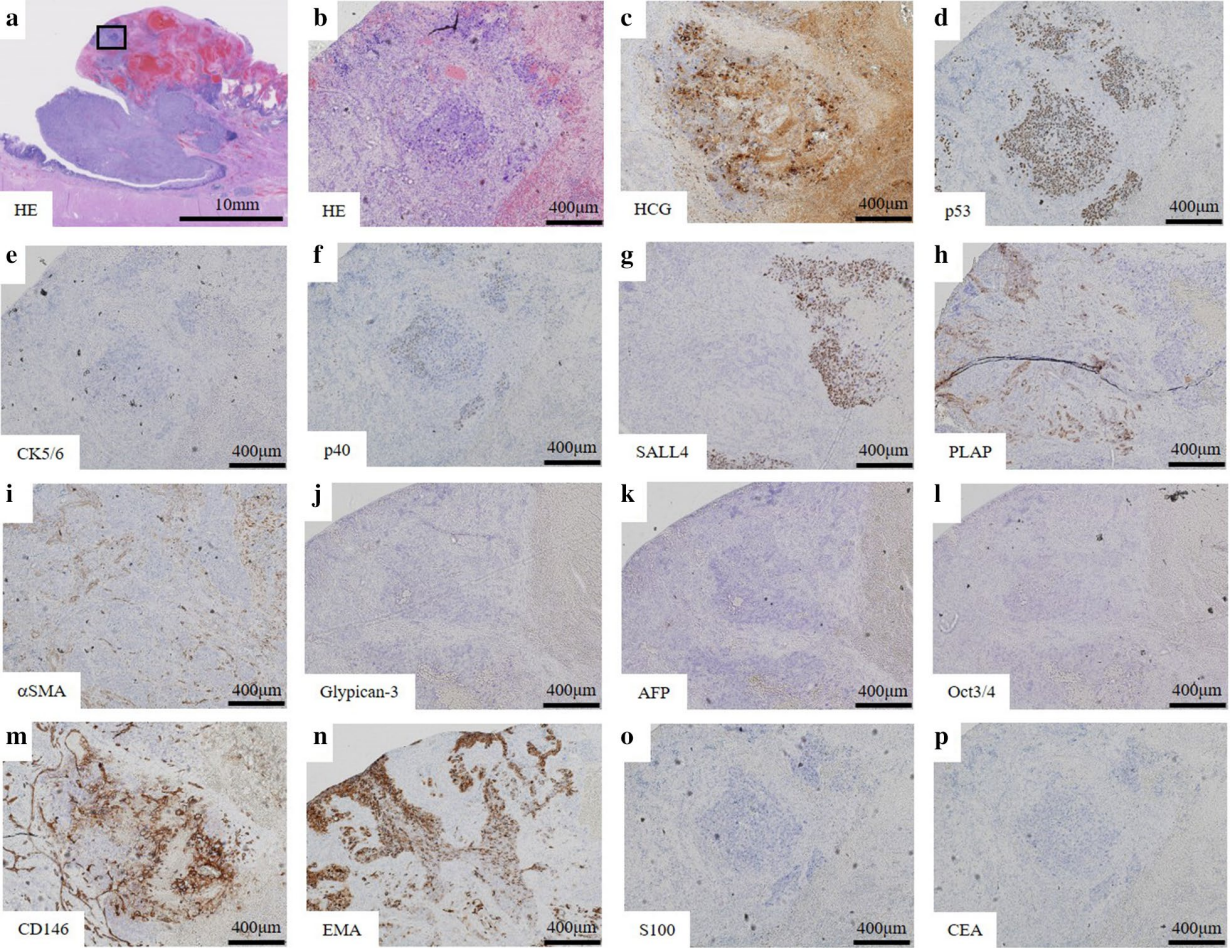
Microscopic and immunohistochemical findings of the choriocarcinoma. High magnification photomicrograph enclosed in a square in **a** is presented in **b**–**n**. The hemorrhagic portion contains large choriocarcinoma cells with syncytiotrophoblasts including condensed nuclei and an acidophilic cytoplasm (**a**, **b**). Strong hCG-positive staining was focally observed in syncytiotrophoblasts (**c**) and these cells were focally positive with p53, p40, SALL4, PLAP, CD146, EMA staining indicating a choriocarcinoma (**d**, **f**, **g**, **h**, **m**, **n**). These large cells were immunohistochemically negative for CK5/6, αSMA, glypican-3, AFP, Oct3/4, S100 and CEA (**e**, **i**, **j**, **k**, **l**, **o**, **p**)

**Fig. 6:**
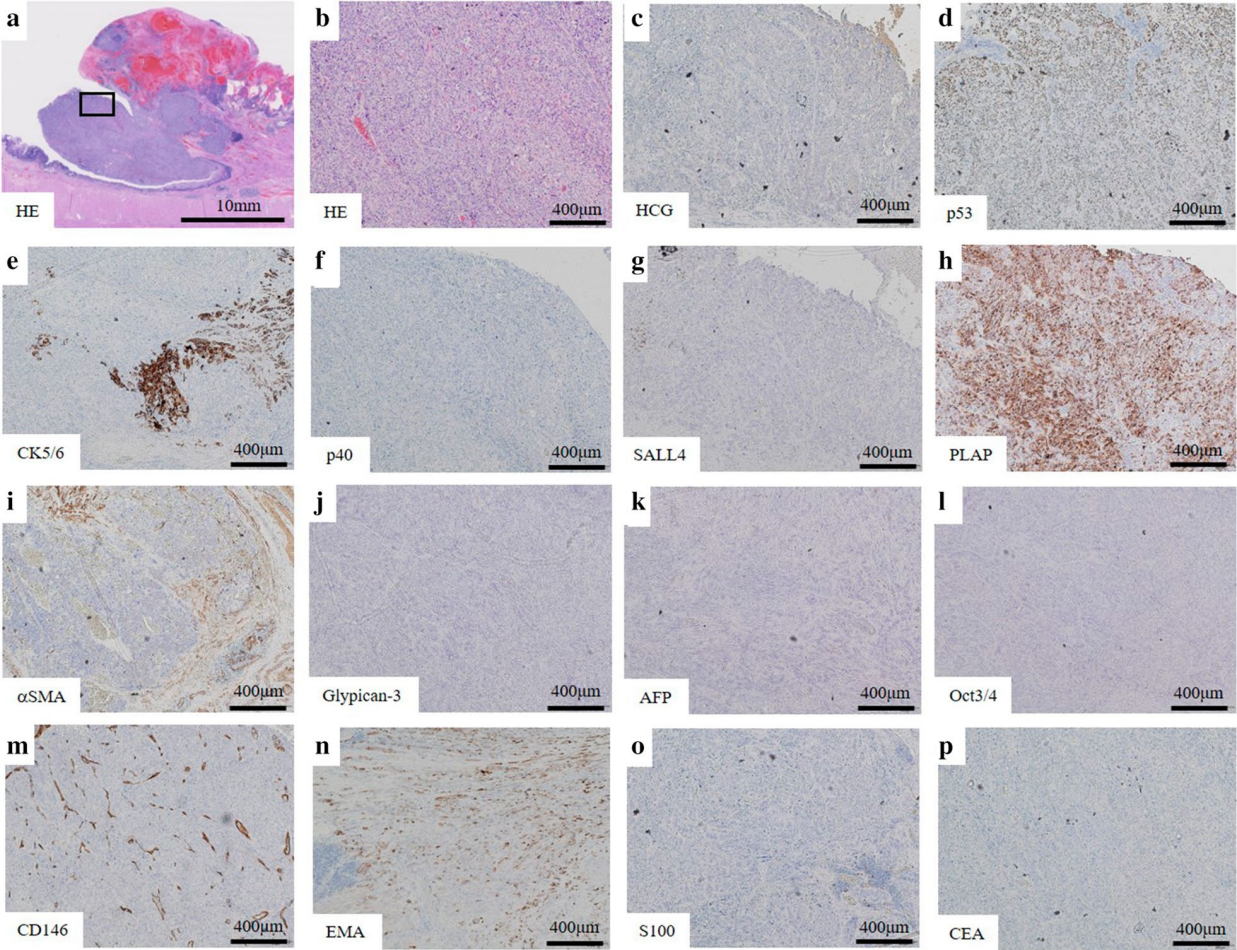
Microscopic and immunohistochemical findings of the choriocarcinoma. High magnification photomicrograph enclosed in a black square in **a** is presented in **b**–**n**. The medullary portion contained small carcinoma cells with a tendency to differentiate into layers (**a**, **b**). These poorly differentiated squamous cell carcinoma-like cell clusters were negative for hCG, p40, αSMA, glypican-3, AFP, Oct3/4, CD146, S100 and CEA (**c**, **f**, **i**–**m**, **o**, **p**) and focally positive for p53, CK5/6, SALL4, PLAP and EMA (**d**, **e**, **g**, **h**, **n**) using immunohistochemical staining

**Fig. 7:**
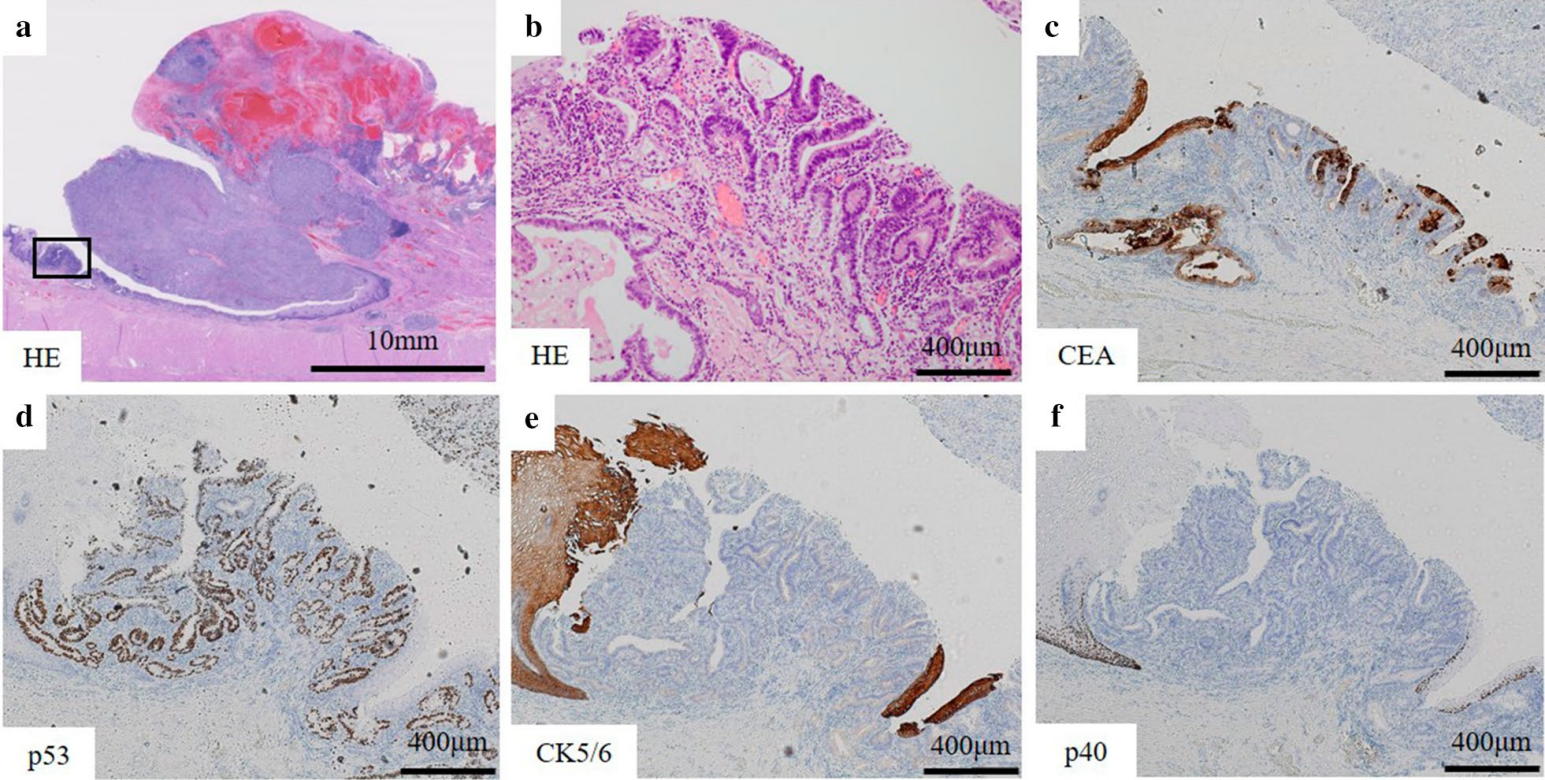
Microscopic findings of the Barrett’s adenocarcinoma. **a** Showed at lower magnification. High magnification photomicrographs enclosed by a black square in **a** are presented in **b**–**f**. A well differentiated adenocarcinoma with CEA and p53-positive clear nuclei in immunohistochemical staining were observed in the Barrett’s epithelium of esophagogastric junction apart from the elevated tumor (black square) (**c**, **d**). These adenocarcinoma cells were negative for CK5/6 and p40 using immunohistochemical staining (**e**, **f**)

Macroscopically, intestinal epithelium indicating LSBE was observed from the middle of the resected esophagus to esophagogastric junction and a protruding tumor 2.5 × 2.3 × 1.0 cm in diameter was found at the oral edge of the LSBE (Fig. 3). Clotting blood and necrotic tissues were attached on the surface of the elevated tumor. Histologically, most of the tumor was accompanied by hemorrhage (Fig. 4a). Most of the elevated tumor was positive for hCG using immunohistochemical staining (Fig. 4b). The tumor comprised large and small carcinoma cells with a high nuclear/cytoplasm ratio. Large cells had a condensed nucleus and an acidophilic cytoplasm, indicative of syncytiotrophoblasts (Fig. 5a, b). These cells were focally positive with hCG, p53, p40, sal-like protein 4 (SALL4), placental alkaline phosphatase (PLAP), CD146 and epithelial membrane antigen (EMA) staining, which indicated a choriocarcinoma (Fig. 5c, d, f–h, m, n). These large cells were immunohistochemically negative for CK5/6, α-smooth muscle actin (αSMA), glypican-3, alpha-fetoprotein (AFP), Oct3/4, S100 and CEA (Fig. 5e, i–l, o, p). Conversely, small carcinoma cells showed a tendency to differentiate into layers (Fig. 6a, b). These cells were negative for hCG, p40, αSMA, glypican-3, AFP, Oct3/4, CD146, S100 and CEA (Fig. 6c, f, i–m, o, p) and focally positive for p53, CK5/6, SALL4, PLAP and EMA using immunohistochemical staining (Fig. 6d, e, g, h, n). From these findings, the tumor contained a carcinoma component similar to a poorly differentiated SCC. However, both large and small carcinoma cells were totally c-Kit-negative, CD34-negative, αSMA-partially positive, PLAP-positive, SALL4-focally positive, AFP-negative, CD30-negative, glypican-3-slightly positive and Oct3/4-negative using immunohistochemical staining. Both large and small carcinoma cells were diagnosed as a choriocarcinoma using immunohistochemical staining. The results of immunohistochemical staining with the typical staining pattern of choriocarcinomas and SCCs are summarized in Table 1. Furthermore, there was a small well-differentiated adenocarcinoma lesion in the Barrett's epithelium on the anal side of the elevated tumor with CEA and p53-positive immunoactivity (Fig. 7a–d). These adenocarcinoma cells were negative for CK5/6 and p40 using immunohistochemical staining (Fig. 7e, f). Finally, the tumor was diagnosed as a primary esophageal choriocarcinoma (pT1b-SM3 INFb ly0 v1 pPM0 pDM pN0 M0 pStage I) in association with Barrett’s adenocarcinoma (Lt 4 mm, pT1a-SMM N0 M0 pStage IA). Thus, the tumor was suggested to be curatively resected.

**Table 1:** Summary of immunohistopathological findings of choriocarcinoma, SCC and our case

Antibodies	Choriocarcinoma	SCC	Large carcinoma cells portion of our case	Small carcinoma cells portion of our case
hCG	++	−	+	Focally+
AFP	±	−	–	–
S100	−	−	–	–
CK5/6	±	+	–	Focally+
Glypican3	±	−	–	–
p40	±	+	Focally+	–
p53	+	+	Focally+	+
Vimentin	±	±	–	–
αSMA	−	−	–	–
PLAP	+	−	Focally+	++
SALL4	+	−	Focally+	Focally+
Oct3/4	+	−	–	–
CEA	−	±	–	–
EMA	±	±	Focally+	Focally++
CD146	±	−	Focally+	Focally+
hPL	±	−	±	–

*SCC* squamous cell carcinoma, *hCG* human chorionic gonadotropin, *AFP* alpha-fetoprotein, *αSMA* α-smooth muscle actin, *PLAP* placental alkaline phosphatase, *SALL4* sal-like protein 4, *CEA* carcinoembryonic antigen, *EMA* epithelial membrane antigen, *hPL* human placental lactogen

## Postoperative course

**Fig. 8:**
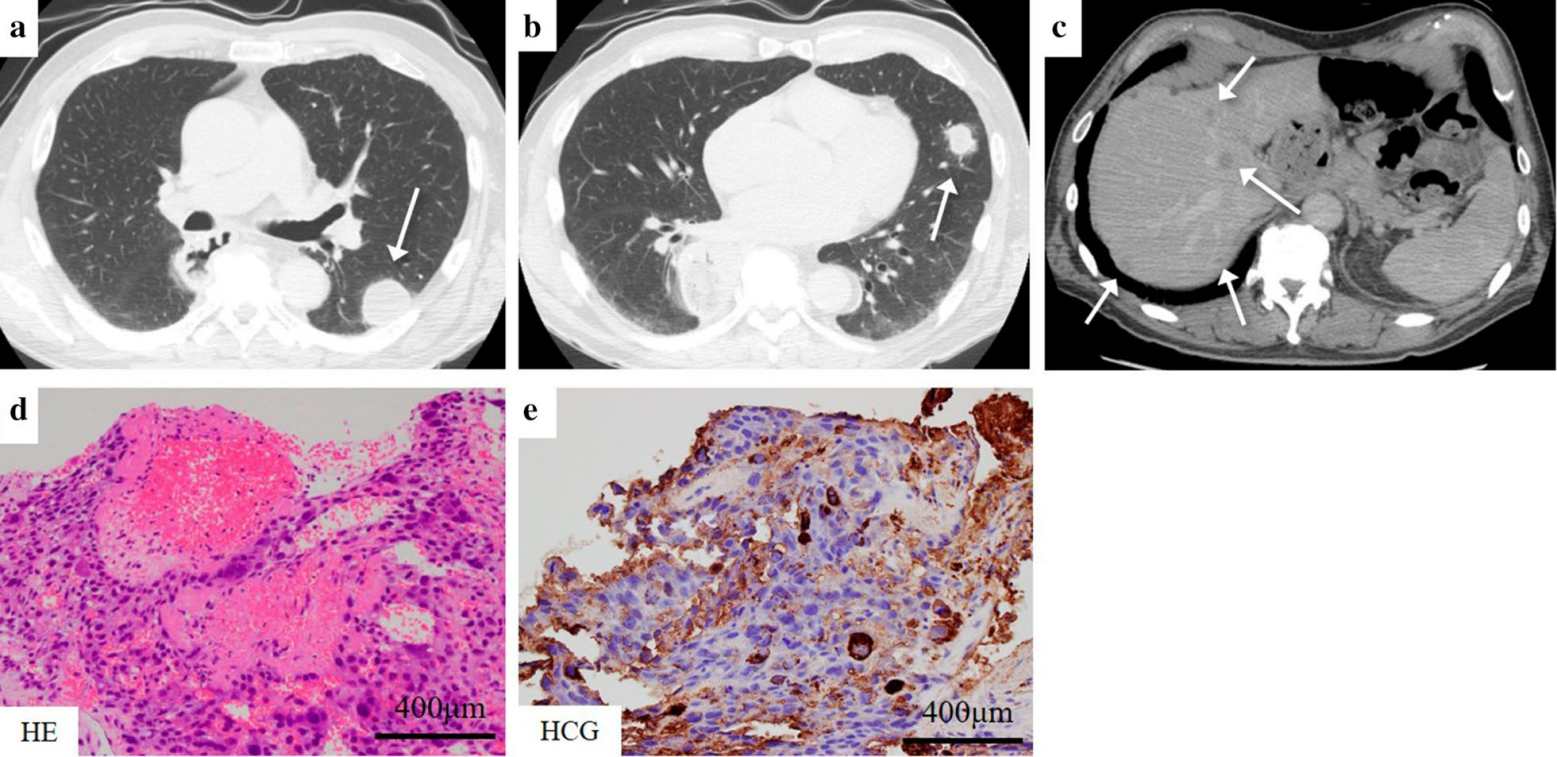
Multiple lung and liver metastatic nodules were detected 3 months after surgery using contrast-enhanced CT examination (**a**–**c**). Tumor cells obtained from the lung nodule using a needle biopsy were similar to esophageal tumors resected previously and expressed hCG using immunohistochemical staining (**d**, **e**)

The patient was discharged on the 28th postoperative day without serious postoperative complications. Three months after surgery, he was readmitted for the treatment of a left renal cell carcinoma. A preoperative CT scan demonstrated multiple lung metastatic nodules and intrahepatic masses (Fig. 8a–c). The value of HCG-β and CYFRA were well above the normal limits (27.8 ng/ml and 3.5 ng/ml, respectively). One of the pulmonary nodules was investigated using a CT-guided needle biopsy. The tumor cells obtained by needle biopsy were similar to those of his esophageal tumors resected previously and expressed hCG and CAM 5.2 and were negative for p40, CK5/6 and thyroid transcription factor-1 (TTF-1) using immunohistochemistry (Fig. 8d, e). There was no germ cell tumor in the testis or brain using testicular ultrasonography and brain magnetic resonance imaging (MRI). Thus, it was diagnosed as multiple lung and liver metastases from a primary esophageal choriocarcinoma. At the same time, a CT-guided needle biopsy was performed on the left renal tumor and a definitive diagnosis of primary renal clear cell carcinoma was made. We decided to administer chemotherapy for the recurrent choriocarcinoma in preference to performing renal resection for left renal cancer.

Chemotherapy for the recurrent choriocarcinoma was performed, employing the regimen for germ cell tumors. The regimen comprised administering three cycles of cisplatin (CDDP) and etoposide (VP-16). CDDP (20 mg/m^2^) was administered on day 1 and VP-16 (100 mg/m^2^) was administered on days 1, 8 and 15 per monthly cycle. After two cycles of CDDP and VP-16, the regimen of chemotherapy was changed. CDDP (20 mg/m^2^/per day) and VP-16 (100 mg/m^2^/per day) were administered on days 1–5 per monthly cycle to reduce chemotherapy-induced adverse events. After three cycles of chemotherapy, metastatic lung and hepatic tumors were tentatively reduced. Afterwards, metastatic tumors regrew rapidly and multiple bone metastases appeared 2 months later. He died of his esophageal choriocarcinoma 13 months after primary resection.

## Discussion

Choriocarcinomas are classified as gonadal choriocarcinomas or extragonadal choriocarcinomas. Extragonadal choriocarcinomas have been reported to occur in testes, pineal gland, lungs, mediastinum and retroperitoneum and its incidence has been reported to be 2–5% of all choriocarcinomas in males [1]. A primary choriocarcinoma of the gastrointestinal tract is rare and even the most frequent primary gastric choriocarcinomas have been reported to arise in approximately 0.08% of stomach malignant tumors [2–4]. Primary esophageal choriocarcinomas are extremely rare and only nine cases worldwide have been reported to have occurred after 1970 [5–13].

According to the definition of a choriocarcinoma, it was needed to recognize eosinophilic and multinucleated syncytiotrophoblast cells in tumor tissues and cytotrophoblast cells with clear cytoplasm and positive immunohistochemical staining of hCG. To diagnose a primary choriocarcinoma of the esophagus, it is necessary to exclude primary sites of other organs including testis, mediastinum, retroperitoneal and pineal gland, where extragonadal choriocarcinomas frequently occur [14]. In cases with necrotic or scar tissue in testicular biopsied specimens, the testes can be the primary lesion, which is called “burned-out-tumor”. However, there is a possibility that only the extragonadal tumor without a naturally regressed primary lesion exists in cases even if scar tissues do not exist in specimens from testes, which could make it extremely difficult to strictly diagnose [15].

The mechanism involved in the development of an esophageal choriocarcinoma is still unknown. Extragonadal choriocarcinomas are thought to occur by a different mechanism compared with gonadal choriocarcinomas. There are several hypotheses related to the occurrence of extragonadal choriocarcinomas: that normal tumor cells retro-differentiate into chorionic cells [16, 17]: that embryonic cells arising in the embryonic phase aberrate [18], that it arises from a common progenitor cell through another differentiation pathway [19] and that the choriocarcinoma occurring in the genitals has metastasized to multiple organs and the primary tumor disappears. In cases of gastric choriocarcinomas, there are many cases combined with adenocarcinomas, so the hypothesis that it arises from retro-differentiation of an adenocarcinoma is most prevalent.

Ten cases of primary esophageal choriocarcinomas including the previously reported nine cases and our case are shown in Table 2 [5–13]. The average age was 57.0 (40–80) years and the age of onset was slightly lower than that of patients diagnosed with an esophageal SCC. The proportion of male cases was about 60%, which indicates little to no gender difference. SCCs and adenocarcinomas were combined in 3 and 5 of 10 cases, respectively. There were only two cases in which only the choriocarcinoma component was observed. There were some reports of a choriocarcinoma combined with an SCC-like component [8, 9]. These reports showed that these SCC-like components were basically choriocarcinomas. Matsukawa et al. and Vallonthaiel et al. have reported that pulmonary choriocarcinoma p40-positive features of cytotrophoblast-like polygonal tumor cells were focally observed in 50% of cells and CK5/6-positive trophoblastic tumor cells could be focally identified in choriocarcinomas. They have exhibited the diagnostic pitfall of primary or metastatic pulmonary choriocarcinomas, which could mimic SCCs morphologically and immunohistochemically [19, 20]. In Table 1, we show the typical pattern of immunohistochemical staining of a choriocarcinoma and an SCC and compare them with the immunohistochemical pattern observed in our case. The portion including large carcinoma cells exhibited positive-hCG, p40, PLAP, SALL4, EMA and CD146, which matched with a typical pattern of the syncytiotrophoblast-like component in choriocarcinomas. Conversely, the portion that comprised small carcinoma cells was morphologically similar to SCCs. These cells exhibited focally positive hCG, apparent positive CK5/6, PLAP, SALL4, EMA and CD146. The expression pattern of these markers in small carcinoma cells was consistent with the pattern of the cytotrophoblast component in choriocarcinomas. In our case, initial histological diagnosis was primary esophageal collision tumor with a choriocarcinoma and an SCC. However, the SCC-like component was finally diagnosed as also a choriocarcinoma using precise immunohistochemical staining. Previously reported cases of esophageal choriocarcinomas combined with SCCs or adenocarcinomas were more likely to be single choriocarcinomas. Precise immunohistochemical staining is necessary to make a correct pathological diagnosis.

**Table 2:** Summary of 10 cases with esophageal choriocarcinomas

Case No	Author	Year	Age	Sex	Location	Histological type	b-hCG	Tumor depth	LN metastasis	Distant metastasis	Treatment	Prognosis
1	Sasano [5]	1970	74	F	Middle-Lower	Choriocarcinoma Adenocarcinoma	Urine	T4	+	Lung Liver	Chemotherapy	4M, dead
2	McKechnie [6]	1971	44	M	Middle-Lower	Choriocarcinoma Adenocarcinoma	Urine	T1b	−	Lung Liver Stomach	Radiation	2M, dead
3	Trillo [7]	1979	40	F	EGJ	Choriocarcinoma	–	T2	−	Lung Pleura Liver Kidney	–	4M, dead
4	Kikuchi [8]	1989	42	M	Middle	Choriocarcinoma	Immunostaining	T4	+	Lung Liver	–	1M, dead
5	Wasan [9]	1993	49	M	EGJ	Choriocarcinoma Yolk sac carcinoma Adenocarcinoma	Serum Immunostaining	–	−	Lung Brain	Chemotherapy	-
6	Motoyama [10]	1995	80	F	EGJ	Choriocarcinoma Hepatoid adenocarcinoma Small cell carcinoma Tubular adenocarcinoma	–	-	−	Lung Liver Stomach	Chemotherapy	2M, dead
7	Merimsky [11]	2000	53	M	Lower	Choriocarcinoma Squamous cell carcinoma	Immunostaining	–	-	Liver Brain	Chemotherapy	8M, dead
8	Ishihara [12]	2002	70	M	Middle	Choriocarcinoma Squamous cell carcinoma Mucoepidermoid carcinoma	Immunostaining	T1b	+	Lung Liver Pleura	–	2M, dead
9	Patil [13]	2006	60	F	Lower	Choriocarcinoma Squamous cell carcinoma	Serum	–	−	Liver	Chemotherapy	–
10	Present case	2018	58	M		Choriocarcinoma Adenocarcinoma	Serum	pT1b	−	–	Operation	14M, dead

*EGJ* esophagogastric junction, *LN* lymph node, *hCG* human chorionic gonadotropin

Many cases showed distant metastasis at the time of first visit. Metastases to other organs were frequently observed in lung, liver and lymph nodes. In our case, although a simultaneous primary renal cancer was combined, distant metastasis and lymph node metastasis of choriocarcinoma were not observed preoperatively and curative resection of the esophageal tumor was possible. First, the tumor was diagnosed as a poorly differentiated carcinoma in the preoperative biopsy. Preoperative chemotherapy might be taken into consideration if we could diagnose it as a choriocarcinoma preoperatively. However, in actuality, we performed a primary resection because of the ease with which the tumor bled and the need for continuation of antiplatelet medicine because of the patient’s cerebral infarction. No case of a curative resection for a primary esophageal choriocarcinoma had been reported previously. Thus, our case was thought to be the first presentation of a curatively resected esophageal choriocarcinoma. Of the past nine cases, half of them were not diagnosed using an endoscopic biopsy beforehand and they were diagnosed as primary esophageal choriocarcinomas at necropsy. In our case, it was also difficult to make an accurate diagnosis from small biopsied specimens. After resection, the resected specimens were immunohistochemically investigated using certain primary antibodies including CK5/6, p40, S-100, CEA, p53, PLAP, SALL4 and hCG, which enabled the diagnosis. Therefore, it is be very important to investigate immunohistochemically using various adequate primary antibodies and to measure germ cell tumor markers preoperatively in cases in which the histopathological diagnosis is difficult using just endoscopic biopsied specimens. In previous reports, most cases of primary esophageal choriocarcinomas with synchronous distant metastases have been treated with systemic chemotherapy. Standard chemotherapy regimens for primary esophageal choriocarcinomas have not yet been established. BEP therapy (bleomycin, etoposide and cisplatin), PE therapy (cisplatin and etoposide), or FP therapy (5-FU and cisplatin) have been selected for the treatment of esophageal SCCs. Wasan et al. reported that complete remission has been obtained with BEP therapy for choriocarcinomas associated with Barrett’s esophageal adenocarcinomas [21]. In all other cases, the survival period was less than 1 year and their prognosis has been reported to be very poor. In our case, PE therapy, which was thought to be effective for the germ cell tumor, was performed for lung and hepatic metastases after the patient relapsed following surgery. The pulmonary and hepatic metastases tentatively reduced after the induction of PE therapy. However, a sufficient survival benefit could not be obtained using chemotherapy. This is the first case of surgically resected choriocarcinoma of the esophagus. Despite surgical resection, early hematogenous recurrence occurred. For improving survival of the patients with esophageal choriocarcinoma, systemic therapy in combination with local control by surgical resection might be necessary. Considering the recent therapeutic strategy of esophageal cancer, preoperative chemotherapy may suppress early postoperative recurrence. If definitive preoperative diagnosis was possible, preoperative chemotherapy followed by surgical resection would be more effective to improve survival.

## Conclusions

In conclusion, we experienced a primary esophageal choriocarcinoma combined with an SCC-like component in association with Barrett’s adenocarcinoma, which could be curatively resected. Primary esophageal choriocarcinoma is a very rare disease with extensive malignant potential. It will be necessary to accumulate additional cases to establish a more effective treatment strategy.

## Data Availability

The patient data for this case report will not be shared to ensure patient confidentiality.

## Abbreviations

CT: Computed tomography
hCG: Human chorionic gonadotropin
SCC: Squamous cell carcinoma
CEA: Carcinoembryonic antigen
CYFRA: Cytokeratin 19 fragment
LSBE: Long segment Barrett’s esophagus
FDG: 2-[18F] fluoro-2-deoxy-d-glucose
PET: Positron emission tomography
SUVmax: The maximum standardized uptake values
SALL4: Sal-like protein 4
PLAP: Placental alkaline phosphatase
EMA: Epithelial membrane antigen
αSMA: α-Smooth muscle actin
AFP: Alpha-fetoprotein
TTF-1: Thyroid transcription factor-1
MRI: Magnetic resonance imaging
CDDP: Cisplatin
VP-16: Etoposide
hPL: Human placental lactogen
EGJ: Esophagogastric junction
LN: Lymph node
